# Quantitative analysis of melanin content in a three-dimensional melanoma cell culture

**DOI:** 10.1038/s41598-018-37055-y

**Published:** 2019-01-28

**Authors:** Soobin Chung, Gippeum J. Lim, Ji Youn Lee

**Affiliations:** 1 Center for Bioanalysis, Division of Chemical and Medical Metrology, Korea Research Institute of Standards and Science, 267 Gajeong-ro, Yuseong-gu, Daejeon, 34113 Republic of Korea; 2 Department of Bio-Analytical Science, University of Science & Technology, 217 Gajeong-ro, Youseong-gu, Daejeon, 34113 Republic of Korea; 3 Department of Biological Sciences, Korea Advanced Institute of Science and Technology, 291 Daehak-ro, Yuseong-gu, Daejeon, 34141 Republic of Korea

## Abstract

Reliable measurement of the amount of melanin produced by melanocytes is essential to study various skin disorders and to evaluate the efficacy of candidate reagents for such disorders or for whitening purposes. Conventional melanin quantification methods are based on absorption spectroscopy, which measures the melanin from lysed cells grown on two-dimensional (2D) surfaces. The 2D culture environment is intrinsically different from *in vivo* systems though, and therefore cells often lose their original phenotypes. Melanocytes in particular lose their ability to synthesize melanin, thereby requiring melanogenesis stimulators such as alpha-melanocyte stimulating hormone (α-MSH) to promote melanin synthesis. In this study, we compared melanin synthesis in B16 murine melanoma cells grown in 2D and three-dimensional culture environments. B16 cells instantly formed an aggregate in a hanging-drop culture, and synthesized melanin efficiently without treatment of α-MSH. We were able to measure the melanin secreted from a single melanocyte aggregate, indicating that our method enables non-invasive long-term monitoring of melanin synthesis and secretion in a high-throughput format. We successfully tested the developed platform by quantifying the depigmenting effects of arbutin and kojic acid.

## Introduction

*In vitro* assays to measure the amount of melanin, a natural pigment and the primary determinant of skin color, are essential in many studies of skin disorders and for whitening purposes. As highlighted in numerous articles, melanin plays a regulatory role in epidermal homeostasis and melanoma behavior^1–3^. To demonstrate the efficacy of substances that regulate melanin synthesis and secretion, diverse platforms are employed: two-dimensional (2D) cell culture models, animal models, and three-dimensional (3D) models including artificial skin (i.e. skin equivalent). The 2D cell culture platform is the most commonly used, where cells adhere to a flat and solid surface and grow as a single layer. This differs from the *in vivo* environment though, as it fails to recapitulate the complex microenvironment of actual tissue. Cells cultured in a 2D environment tend to exhibit different phenotypes as well as different responses to external stimuli, such as drugs, compared with cells cultured in a 3D environment^4^. Animal models mainly employ mice in skin biology and skin cancer research because their skin is more similar to human skin than other animals^5^; despite this similarity though, mice and human systems are still quite divergent^4,6,7^.

Therefore, the 3D culture model has arisen as an attractive candidate to overcome the drawbacks of both the 2D model, which lacks *in vivo* context, and the animal model, which cannot faithfully reproduce the human system and possesses ethical issues. Three-dimensional cell culture models include explantations of tissues, artificial skin, and cellular spheroids^8,9^. Artificial skin is an *in vitro* model having a structure and characteristics similar to real skin tissue. Since it exhibits reactions similar to those occurring in the body when exposed to external stimuli, it is becoming a popular *in vitro* model to replace animal models. However, artificial skin is expensive, and its composition and construction has not yet been standardized. In addition, it is not yet available in the 96-well plate format and consequently, is not suitable for high-throughput screening. From this point of view, cellular spheroids or aggregates present opportunities for an affordable screening platform to overcome the disadvantages of artificial skin.

A cellular spheroid is a 3D cell culture model based on the principle that cells in suspension assemble and form aggregations by cell−cell adhesion when cell−surface interaction is prevented. Such aggregates can be produced by simple methods without special techniques or equipment, with large scale culture possible^10,11^. Thus, the spheroid model can be useful in screening candidate substances for pigmentation or depigmentation prior to using costly and time-consuming artificial skin or animal models.

For melanin in particular, diverse analytical methods have been employed for quantification, including fluorescence spectrophotometry^12^, electron paramagnetic resonance spectrometry^13–15^, high performance liquid chromatography^16^, and absorption spectroscopy^17–19^. Among them, absorption spectroscopy is the most widely used method because the experimental procedure is simple, and does not require bulky equipment or skilled operators. However, as cells are dissolved in a strong basic solvent at high temperature prior to measurement, this method is destructive and can only be used for end-point measurements^19,20^. In response to this issue, a method for culturing cells in a microplate has been proposed^18,21^, where extracellular melanin secreted into the cell culture medium is non-invasively measured. Both the recording of extracellular melanin levels and the observation of changes in cellular morphology or confluency can be done over time. Further, at the end of the culture, cells can be used to perform additional assays, such as measuring the amount of melanin existing in the cell pellet or measuring the activity of enzymes related to melanogenesis.

In this study, we developed an *in vitro* assay for melanin measurement by combining a 3D cell culture with non-invasive absorption measurement in a well-plate format. We first investigated melanin production by melanocytes in conventional 2D cultures, and then in 3D culture. Finally, we quantitatively measured the inhibitory effect of a few depigmenting agents on the production of melanin using the developed assay.

## Results and Discussion

### 2D monolayer melanocyte culture and the effect of α-MSH treatment on melanocytes

Before exploring the melanin production by melanocytes in a 3D culture, we investigated the melanogenesis characteristics of our cell lines in a conventional 2D monolayer culture. B16 cells are melanoma cell lines derived from C57BL/6 mice^22^, which produce melanin and display metastatic behaviors. Thus, B16 cells are widely used to study melanogenesis and depigmentation^23^, tumor metastasis^24^, and for cytotoxicity measurements of various substances in skin models^25^. In a 2D culture, B16 cells gradually lose their ability to synthesize melanin as subculture continues^26,27^. The cells should therefore be pigmented in advance to be used for depigmentation studies; alpha-melanocyte stimulating hormone (α-MSH) is one of the most commonly employed reagents for pigmentation. It activates adenylate cyclase to escalate the level of cyclic adenosine monophosphate inside of the melanocytes, thereby promoting melanocyte growth and inducing melanogenesis^28,29^. The nonessential amino acid L-tyrosine contained in most cell culture media may stimulate melanogenesis in a hormone-like manner^30^; however, this stimulation by L-tyrosine is insignificant compared to that by α-MSH.

**Figure 1:**
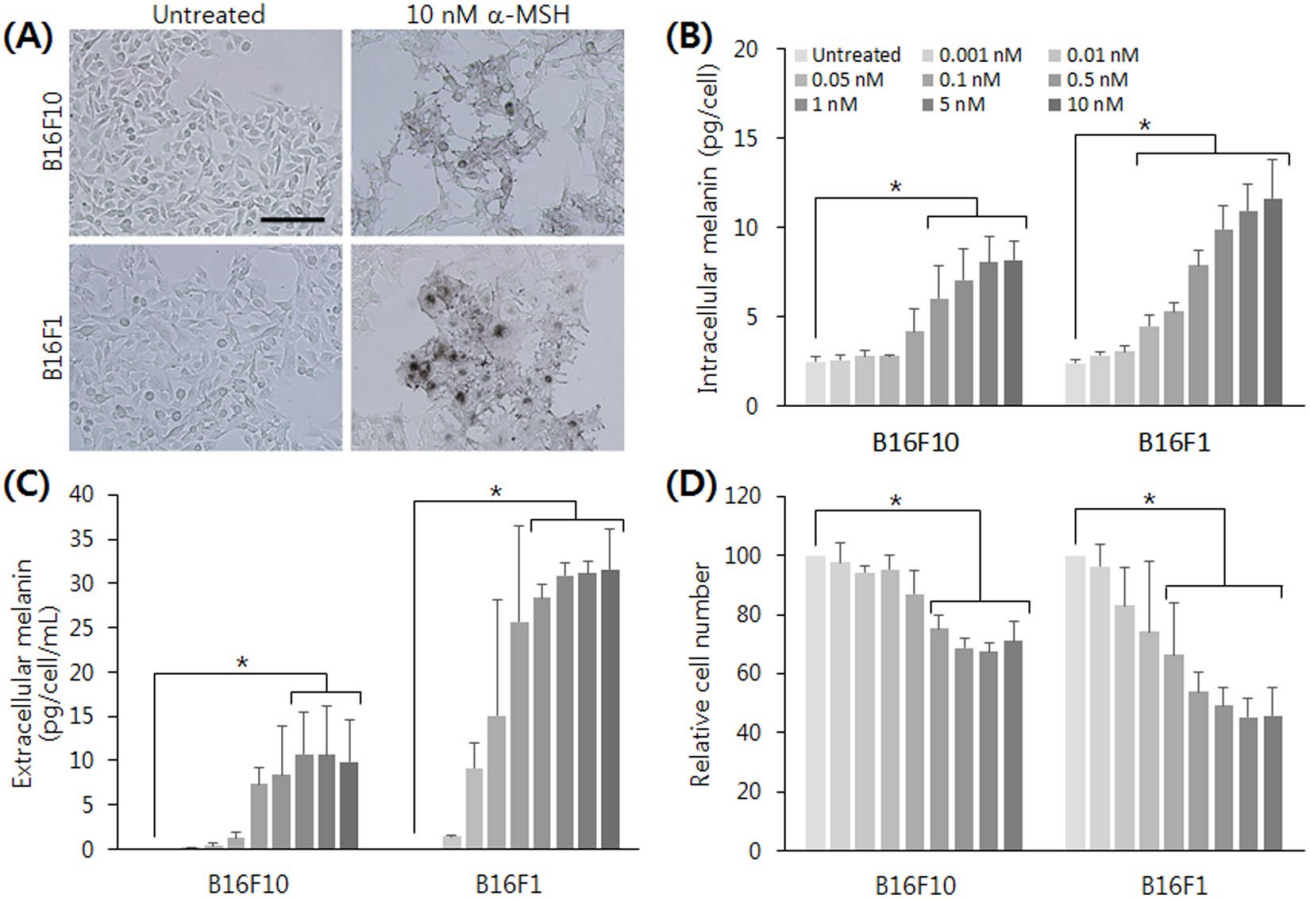
Effect of α-MSH treatment on melanin synthesis and proliferation of 2D cultured melanocytes. α-MSH-induced melanin synthesis of 2D cultured B16 cells. (**A**) 10 nM of α-MSH treatment for 72 hours induced noticeable melanogenesis in both cell lines. Typical dendritic morphology and dark pigmentation were observed. Scale bar = 200 μm. The amount of (**B**) intracellular and (**C**) extracellular melanin showed a dose-dependent response in both cell lines. (**D**) The proliferation of B16 cells was inhibited by α-MSH treatment. The significant difference was analyzed by t-test (*p < 0.05).

With reference to values from previous studies, commonly used B16 cell lines B16F10 and B16F1 were treated with α-MSH ranging from 10 to 50 nM. Upon α-MSH treatment, the cellular shape changed dendritically and the color of the cell pellet, which initially appeared white to gray, turned brown to black, indicating melanin synthesis (Fig. 1A). Such dendritic extensions of melanocytes are known to be essential for the transfer of melanosomes from melanocytes to surrounding keratinocytes^31,32^. The treatment of α-MSH significantly inhibited cellular proliferation, and the amount of melanin did not increase by increasing α-MSH concentration (Fig. S1, Supplemental Information). We then performed the same experiment using a lower range of α-MSH concentrations, from 0.001 to 10 nM. In this range, both intra- and extracellular melanin increased in a dose-dependent manner in both B16F10 and B16F1 cells (Fig. 1B,C). In the case of B16F1 cells, as low as 0.01 nM of α-MSH induced a noticeable level of melanin synthesis. The half maximal effective concentration (EC50) of α-MSH for both cell lines was calculated in the range of 100 to 400 pM. Siegrist *et al*. also reported that B16F10 cells showed dose-dependent increase of extracellular melanin when they were treated with α-MSH in a concentration range of 0.001 to 1 nM, with an α-MSH EC50 of 27 pM^21^. The inhibition of cell growth by α-MSH was also observed in a dose-dependent manner (Fig. 1D). High concentrations of α-MSH (50–100 nM) might be preferred in depigmenting studies to demonstrate a dramatic effect of candidate substances without considering other effects such as proliferation inhibition.

While the level of extracellular melanin in control melanocytes without α-MSH treatment was negligible, the amount of measured intracellular melanin was substantial (several picograms per cell). This raised the question of whether the determined value originated from the intracellular melanin or from other cellular substances in the lysate: when we measure the absorbance from a pellet, we measure the whole lysate, which contains diverse cellular matter including proteins and lipids. The turbidity from those components may contribute to the absorbance, and therefore the melanin amount can be overestimated. To answer this question, we measured the absorbance of cell lysate from a few different cell lines that do not produce melanin. Most of the tested cells showed a similar or lower level of absorbance to unpigmented B16F10 and B16F1 cells (Fig. S2, Supplemental Information), but one tested line (MCF7) showed a considerable level of absorbance. We suppose that such high absorbance of MCF7 mainly resulted from other cellular matter, because the absorbance decreased significantly after centrifugation, unlike the case of B16 cells. When we measure relative parameters such as EC50, the background absorbance by cellular substances can be ignored. However, the level of background absorbance should be considered when the absolute quantification of melanin is being sought. This phenomenon can be overcome by using a real sample-like standard curve, for example a melanin spike-in to a cell lysate. We also note that, for a better quantification, one should consider using natural melanin to prepare a standard curve because calibration with synthetic melanin may cause a systematic bias in measuring natural melanin in culture.

### 3D melanocyte culture and non-invasive measurement of melanin

**Figure 2:**
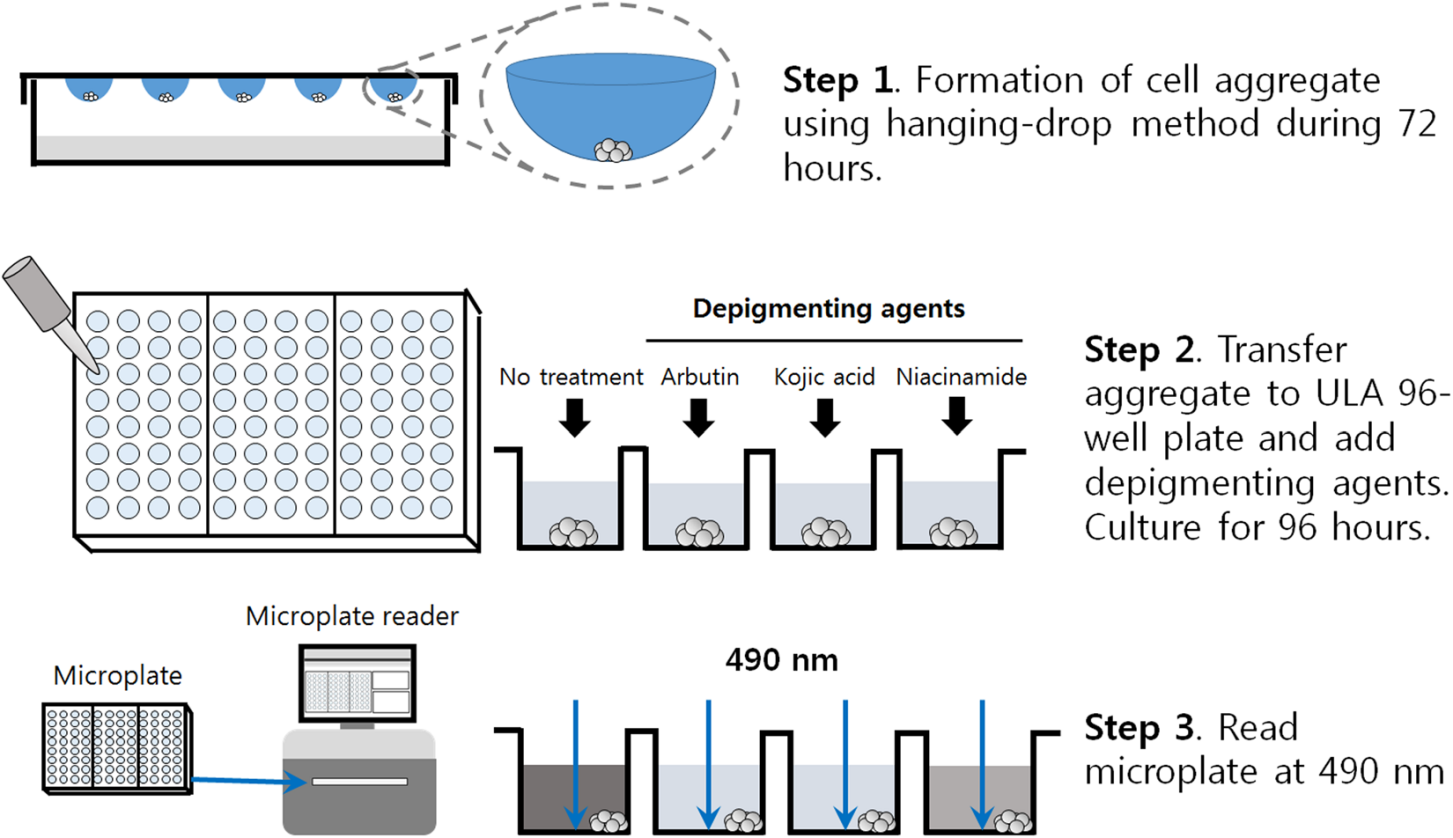
Schematic overview of the developed method for a non-invasive measurement of extracellular melanin from 3D-cultured melanocytes. (Step 1) Melanocytes are allowed to form an aggregate in a hanging drop for 72 hours. (Step 2) The aggregates are transferred to a ULA flat-bottom 96-well plate with or without the addition of depigmenting agents. (Step 3) The melanin present in cell culture medium is monitored by measuring the absorbance at 490 nm for 4 days.

**Figure 3:**
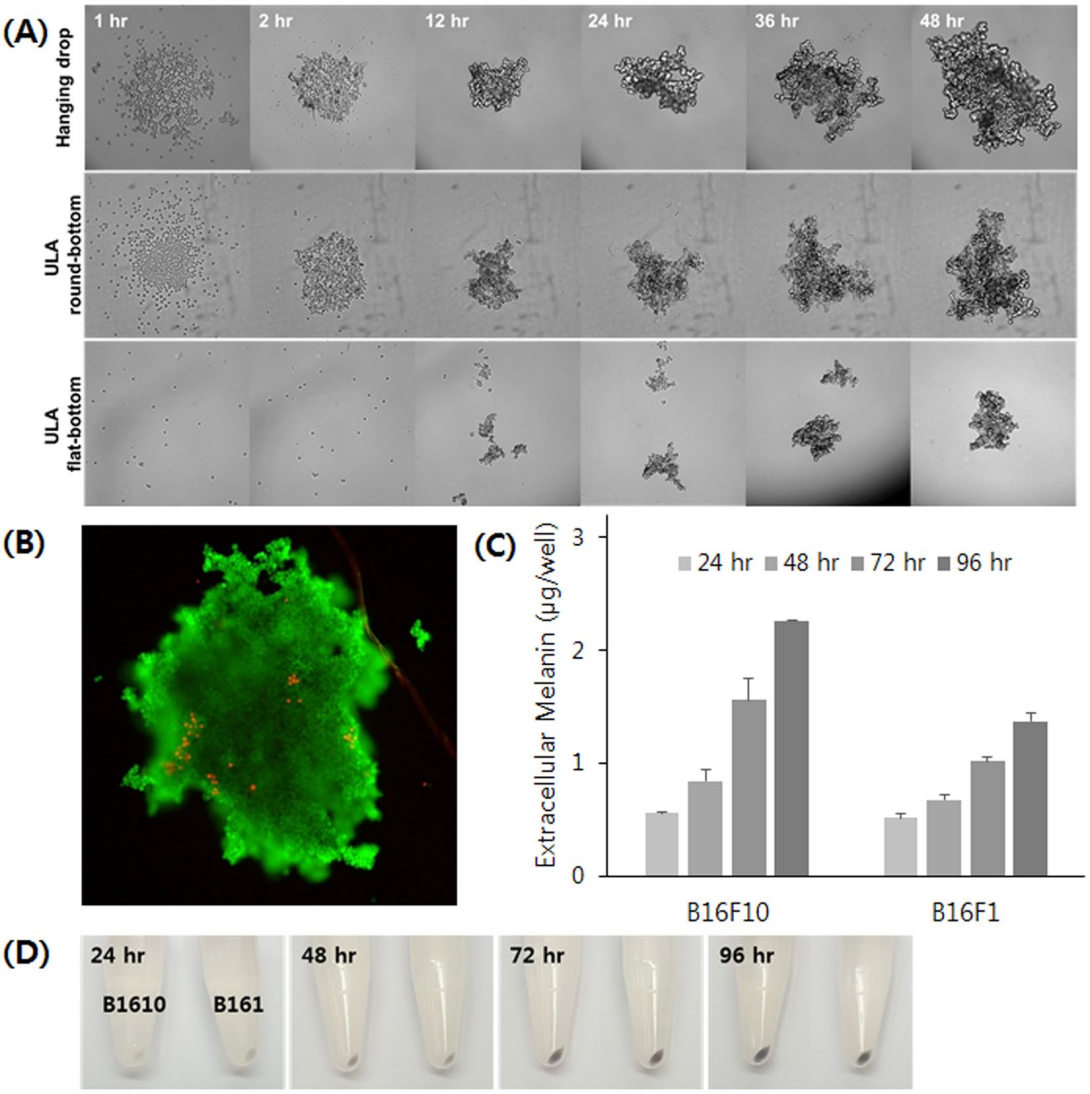
3D culture of melanocytes and their spontaneous melanin production. (**A**) B16F10 cells formed a single aggregate in a hanging-drop and a ULA round-bottom plate, but they formed a few small aggregates in a flat-bottom plate. (**B**) Live/dead staining of a B16F10 aggregate demonstrates that most of the cells are live after 72 hours of culture in a hanging drop. (**C**) Melanocyte aggregates in a 3D culture produced melanin without the addition of melanin-synthesizing hormones. (**D**) A pellet of melanocyte aggregate demonstrated the increase of melanin production by time.

In our study, we made an aggregate of B16 cells by the hanging-drop method as described in Fig. 2. The process of aggregate formation and growth was recorded by time-lapse live imaging (Fig. 3A, and Supplemental Video). Initially, B16F10 cells were scattered in the hanging drop before gradually moving to the lower side of the drop due to gravity. Adhesion among cells progressed to form an aggregate in about 12 hours, after which it grew continuously. Most of the cells in the aggregate were live at 72 hours in culture (Fig. 3B). Interestingly, when we transferred the aggregates to a Petri dish for a prolonged culture in suspension, the culture medium appeared dark brown in color after a few days even without the presence of α-MSH, an observation also reported in other literature^33^. Since the addition of α-MSH can affect the cellular status (e.g. proliferation inhibition), such spontaneous melanin production by cellular aggregation formation is a very attractive characteristic.

For high-throughput screening, a 96-well format is preferred over conventional dish or flask cultures. First, cells grown in 3D can easily achieve a high cell density. Also, the level of extracellular melanin in 3D-cultured cells was high enough to be detected with the naked eye, meaning that we can measure the extracellular melanin without destroying cells. Therefore, 3D melanocyte culture in a 96-well plate format opens up the possibility of non-invasive high-throughput measurement of melanin. With this culture, we systematically investigated melanocyte aggregate formation and quantitatively measured melanin production.

First, we tested different initial cell numbers for the aggregate formation. B16F10 cells of all tested initial cell numbers (500, 1,000 and 2,000) successfully formed aggregates (Fig. S3(A), Supplemental Information). While melanocytes of higher initial cell number generated a larger aggregate and produced higher levels of melanin, the media in the drop acidified faster concurrently. Thus, we decided to use 1,000 cells initially. The melanocyte aggregate grew as a sheet several hundreds of microns thick, rather than as a spherical compact where the supply of oxygen and nutrition is limited. Therefore, the size of B16 aggregates continuously increased over time without noticeable retardation until 6 days (Fig. S3(B), Supplemental Information). After 72 hours, the average diameter of the aggregate was about 681 ± 28 μm in the case of B16F10 cells. B16F1 cells showed similar behavior in the process of cellular aggregation, but the growth rate was slower than that of B16F10 cells, as similarly observed in 2D culture (data not shown).

Although the hanging-drop method is an efficient and inexpensive way to produce a cellular aggregate, it has a few limitations. A long-term culture is not feasible because of the small volume of the drop, which is usually 20 to 30 μL. The drop is also prone to evaporation and pH change, and washing or treating reagents cells in the drop is not straightforward. Therefore, we transferred the aggregate to an ultra-low attachment (ULA) flat-bottom 96-well plate after 72 hours and continued the culture for an additional 96 hours. A larger culture volume (200–250 μL) accommodates an extended culture time, and the well-plate format provides the potential for high-throughput screening. In tests using ULA plates from the beginning (Fig. 3A), in the case of a flat-bottom plate, a few small aggregates of different sizes were generated, which were not applicable to our assay. A round-bottom plate was suitable for generating an aggregate of uniform size, however the shape of the plate bottom caused a few problems elsewhere. The center-location of the aggregate hindered measurement of extracellular melanin, and the poor optical transparency was inappropriate for reproducible absorbance measurements. Therefore, the combination of the hanging-drop method with suspension culture in a ULA flat-bottom plate can be the most efficient way to cultivate melanocytes in 3D and to measure the melanin continuously over the course of the culture.

Next, we explored the synthesis and secretion of melanin by melanocyte aggregates. The extracellular melanin was measured at 24-hour intervals (Fig. 3C). As measuring the cell number and culture volume of each 3D aggregate was infeasible, we used a distinct scale for 3D aggregate melanin production (µg/well). Although color change in the culture media was not noticeable until 48 hours post transfer (PT) to the ULA 96-well plate, the amount of melanin per well continued to increase over time. The color of the pellet also darkened over time, indicating active melanin synthesis (Fig. 3D). The amount of extracellular melanin also increased over time to reach 2.26 and 1.37 μg per well from B16F10 and B16F1 cells, respectively, at 96 hours PT. The variation in measurement values between technical quadruplicates was relatively small, with a relative standard deviation lower than 15%.

We then investigated whether the addition of α-MSH further induces melanogenesis in melanocytes. The amount of extracellular melanin significantly increased in a dose-dependent manner from 48 hours PT (Fig. S4, Supplemental Information). At 96 hours PT, the extracellular melanin from 1 nM α-MSH-treated B16F10 cells was about nine times higher than that of untreated cells. In the case of B16F1 cells, the enhancement was much more prominent, as we also observed in 2D culture. Although α-MSH can further induce melanin production, the aggregates from both B16F10 and B16F1 cells produced enough melanin to be detected without the stimulation. The culture time required to accumulate enough melanin to be measurable was similar with and without treatment. Most of all, α-MSH was found to inhibit the proliferation of melanocytes even at a low concentration in our 2D study. Therefore, the depigmenting study described below was conducted without the α-MSH treatment.

### Measurement of depigmenting effects in 3D culture with non-invasive absorption spectroscopy

Depigmenting agents are largely categorized into (1) substances involved in controlling the melanin synthesis process (e.g. controlling the amount or activity of tyrosinase), (2) substances that interfere with the transfer or dispersion of the melanosome, which is a melanin-containing organelle, and (3) substances that promote the turnover of skin cells^34,35^. Here, we tested three well-known depigmenting agents: arbutin, kojic acid, and niacinamide^36^. Arbutin is a β-D-glucopyranoside of hydroquinone, which serves as a competitive inhibitor for tyrosinase, a key enzyme in the melanogenesis pathway which inhibits the maturation of melanosomes. Kojic acid (5-hydroxy-2-hydroxymethyl-4H-pyran-4-one) is a copper chelator and one of the most intensively studied tyrosinase inhibitors. Niacinamide (vitamin B3, nicotinamide, 3-pyridinecarboxamide) is a biologically active form of niacin which interferes with melanosome transfer from melanocytes to keratinocytes.

**Figure 4:**
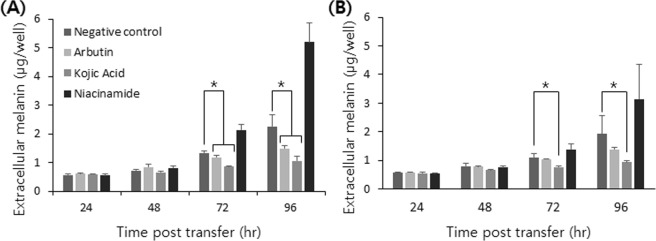
Depigmenting effects of arbutin, kojic acid, and niacinamide on melanocytes in a 3D culture. Time-course change of extracellular melanin production in (**A**) B16F10 and (**B**) B16F1 cells. The significant difference was analyzed by t-test (*p < 0.05).

Based on published depigmenting studies that employed depigmenting agents in a 100 to 1,000 μM range^5,37–39^, we chose a 500 μM concentration to demonstrate the developed assay, a concentration range known to be not cytotoxic^40^. Depigmenting agents were added at the time of transfer of melanocyte aggregate to the 96-well plate. We monitored the morphology of cellular aggregates (Fig. S5, Supplemental Information) and the level of extracellular melanin (Fig. 4). There were no significant differences in size and morphology of aggregates following treatment of depigmenting agents, and both cell lines showed a similar trend in depigmentation. The depigmenting effect of arbutin and kojic acid appeared from 72 hours PT, and became more evident at 96 hours PT, with kojic acid displaying a more pronounced effect of depigmentation. For an easier comparison between samples, we defined a *depigmenting index* (DI) as the percentage of decreased melanin in treated samples to an untreated control, as described in the equation below, with data summarized in Table 1.

$${\rm{Depigmenting}}\,{\rm{index}}\,({\rm{DI}},\, \% )=\frac{Melani{n}_{untreated}-Melani{n}_{treated}}{Melani{n}_{treated}}\times 100$$

**Table 1:** Depigmenting index of arbutin, kojic acid, and niacinamide obtained from 3D cultured B16 cells.

	B16F10			B16F1		
	Arbutin	Kojic acid	Niacinamide	Arbutin	Kojic acid	Niacinamide
Time PT (hr)	DI (%) of extracellular melanin					
72	11.22	36.10	−59.42	5.45	30.65	−25.20
96	34.39	53.57	−129.37	27.76	51.60	−64.11
DI (%) of intracellular melanin						
72	33.03	28.72	−18.67	30.43	−0.95	−25.68
96	36.88	27.98	1.27	26.15	14.08	−4.69

DI was slightly higher in B16F10 cells in general. Kojic acid was the most effective depigmenting agent among tested substances at a concentration of 500 μM, a result consistent with a previous study where kojic acid was more effective than arbutin at a concentration of 500 μM^38^. Niacinamide did not display any depigmenting effect: since niacinamide is involved in the transfer of melanosomes to keratinocytes, it is known that it does not exhibit a depigmenting effect in an environment in which melanocytes are cultured alone^31^. Interestingly, niacinamide rather significantly increased the level of extracellular melanin after 72 hours PT.

We measured the extracellular melanin from a spheroid lysate at the end of the culture to compare the developed method to the conventional one (Fig. S6, Supplemental Information). The result was similar in that both arbutin and kojic acid displayed a depigmenting effect while niacinamide did not. However, in conventional melanin measurement, arbutin appeared to be more effective than kojic acid, and niacinamide did not enhance the intracellular melanin production at all. Explaining this discrepancy is out of our current scope, but we suggest that the potential differences between the intra- and extracellular melanin measurements should be considered.

**Figure 5:**
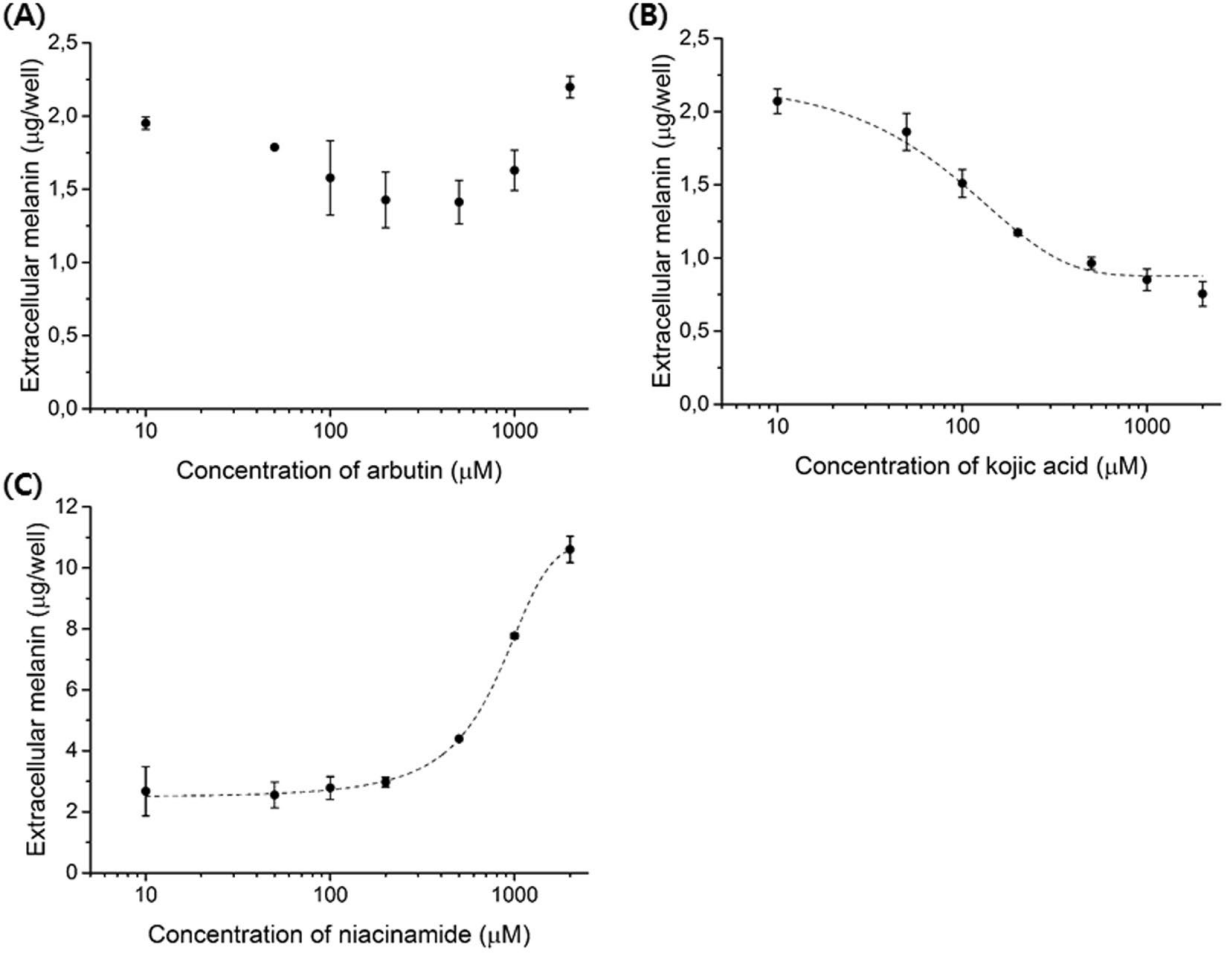
Dose-response of tested depigmenting agents in B16F10 aggregates at 96 hours PT. (**A**) The amount of extracellular melanin did not significantly change with different concentrations of arbutin. (**B**) Kojic acid showed a typical dose-dependent depigmentation. (**C**) Niacinamide concentrations higher than 500 μM significantly induced melanin production.

Lastly, we investigated the melanin production of B16 cell aggregates when they were treated with 10 to 2,000 μM of each depigmenting agent. While arbutin did not show a dose-dependent response in the extracellular melanin, kojic acid and niacinamide did, albeit in a very different direction from 72 hours PT (Figs 5 and S7, Supplemental Information). Kojic acid showed a typical sigmoidal dose-dependent response, and the EC50 for B16F10 cells was calculated to be 115.8 ± 8.0 μM. Niacinamide did not affect melanin production in the range of 10 to 200 μM, however concentrations higher than 500 μM induced a high level of melanin production in a dose-dependent manner. The size and morphology of the B16 aggregates did not change up to 500 µM of each depigmenting agent (Fig. S8, Supplemental Information). However, at concentrations higher than 1,000 µM, the B16 aggregates were considerably smaller in size and more compact in morphology. Therefore, a concentration lower than 500 µM is recommended for the tested depigmenting agents in 3D cultured B16 cells.

In summary, we developed a non-invasive melanin quantification method based on a 3D culture of B16F10 and B16F1 cells, which can be potentially used for a high-throughput screening of depigmenting agents. The method combined a 3D melanocyte aggregation from a hanging-drop array with a suspension culture in a ULA 96-well plate. B16 melanocytes restored their melanin production ability by simply forming an aggregate without the addition of stimulant. We could measure the depigmenting effect of a few depigmenting agents non-invasively using a single melanocyte aggregate. As a result, the melanocyte aggregate can be used as an alternative to the artificial skin model, and fill the gap between the experimental stages of 2D and animal models. We envision that the developed platform can be employed to enable a high-throughput screening of substances involved in the melanin synthesis process of melanocytes.

## Methods

### 2D melanocyte culture and melanogenesis induction by α-MSH treatment

The murine melanoma cell lines B16F10 and B16F1 (ATCC) were cultured in Dulbecco’s Modified Eagle Media (DMEM) supplemented with 10% fetal bovine serum, 100 U/mL penicillin, and 100 μg/mL streptomycin. DMEM without phenol red was used for melanin measurements because phenol red, a pH indicator added to culture medium, can interfere with absorbance measurements. Cells were cultured in a humidified incubator in which 5% CO_2_ was supplied and maintained at 37 °C. All cell culture supplies were purchased from ThermoFisher Scientific unless otherwise indicated. Alpha-melanocyte stimulating hormone (Sigma-Aldrich) was used to induce melanin synthesis in melanocytes. A stock solution of 0.5 mM of α-MSH was prepared in deionized water, and then diluted in a phenol red-free cell culture medium to final concentrations. Melanocytes were incubated for 24 hours at an initial concentration of approximately 1.8 × 10^3^ cells/cm^2^. After 72 hours, the cell pellet and the culture medium were collected. The cell number was estimated from a cell counting using an automatic cell counter (Countess, Invitrogen).

### 3D melanocyte culture and characterization of melanocyte aggregates

The hanging-drop method was used to create melanocyte aggregates. Methylcellulose was added to the cell culture medium at a concentration of 0.24% w/v to make a cell suspension containing an appropriate number of cells. An array of 25 μL drops of culture medium containing 500 to 2,000 cells was made on the lid of the culture dish. Then the lid was carefully flipped over and placed on the top of the 1× PBS-filled culture dish to minimize evaporation of the drop. After 72 hours in a hanging-drop culture, each aggregate was collected and transferred to each well of a ULA flat-bottom 96-well microplate (Corning) using a low-binding tip with a special coating to prevent the aggregate from sticking to the pipette tip. We used a compact imaging device (Juli Br, NanoEnTek) which can be accommodated in a conventional CO_2_ incubator to record the formation and growth of melanocyte aggregates. Time-series images were obtained at intervals of 30 minutes for 72 hours. For live/dead cell staining, melanocyte aggregates were washed once with 1× PBS and labeled with calcein AM and ethidium homodimer-1.

### Treatment of depigmenting agents

Arbutin, kojic acid, and niacinamide were purchased from Sigma-Aldrich, dissolved in deionized water at 20 mM, and stored at −20 °C until use. At the time of use, each depigmenting agent was diluted to an appropriate concentration in a phenol red-free cell culture medium. In the case of monolayer culture, depigmenting agents were added along with α-MSH. In the case of 3D culture, the depigmenting agents were added to the cell culture medium when melanocyte aggregates were transferred to the well plate from hanging-drops at 72 hours after the cell seeding. Then the melanocyte aggregates were exposed to depigmenting agents for an additional 96 hours.

### Measurement of the amount of melanin

When measuring the intracellular melanin from a cell pellet, 100 μL of 1 N NaOH containing 10% DMSO was added to the pellet and heated at 80 °C for 90 minutes. Absorbance was then measured at 490 nm using an EnSpire multimode plate reader (Perkin Elmer). To convert the absorbance value to the amount of melanin, a standard curve was obtained from 0 to 500 μg/mL of synthetic melanin (Sigma-Aldrich) solution dissolved in 1 N NH_4_OH. When measuring the extracellular melanin in 2D culture, 200 μL of the cell culture medium was transferred to a 96-well plate and the absorbance was read. In the case of 3D culture, the culture medium in the well plate was read directly by a plate reader. To avoid possible interference with absorbance by a melanocyte aggregate being present in the path of the light, the aggregate was positioned on the edge of the well by slightly tilting the microplate. Measurement was performed after confirming the relocation of cell aggregates to the edge of the well. We used a standard curve obtained from synthetic melanin dissolved in 1 N NH_4_OH, diluted in a culture medium. The absorbance was averaged from three wells, and each experiment was performed in duplicate or triplicate.

### Statistical analysis

Experiments were independently repeated at least in triplicate. Error bars in the graphical data represent standard deviations. A two-tailed t-test was used for statistical analysis using Excel 2013 (Microsoft), and statistical significance was claimed when the p-value was lower than 0.05.

## Supplementary information

Supporting information

Supporting information video 1

Supporting information video 2

## Data Availability

The datasets generated during the current study are available from the corresponding author on reasonable request.
