# Supplementary material for: Quantitative analysis of melanin content in a three-dimensional melanoma cell culture
Source: Sci Rep. 2019 Jan 28;9:780. doi: 10.1038/s41598-018-37055-y (PMC6349835; doi:10.1038/s41598-018-37055-y)
Supplement: Supplementary file 1 — Supporting information [file 41598_2018_37055_MOESM1_ESM.docx]

**Supplementary Information**

**Quantitative analysis of melanin content in a three-dimensional melanoma cell culture**

Soobin Chung^1,2^, Gippeum J. Lim^3^, Ji Youn Lee^1,*^

^1^ Center for Bioanalysis, Division of Chemical and Medical Metrology, Korea Research Institute of Standards and Science, 267 Gajeong-ro, Yuseong-gu, Daejeon 34113, Republic of Korea

^2^ Department of Bio-Analytical Science, University of Science & Technology, 217 Gajeong-ro, Youseong-gu, Daejeon 34113, Republic of Korea

^3^ Department of Biological Sciences, Korea Advanced Institute of Science and Technology, 291 Daehak-ro, Yuseong-gu, Daejeon 34141, Republic of Korea.


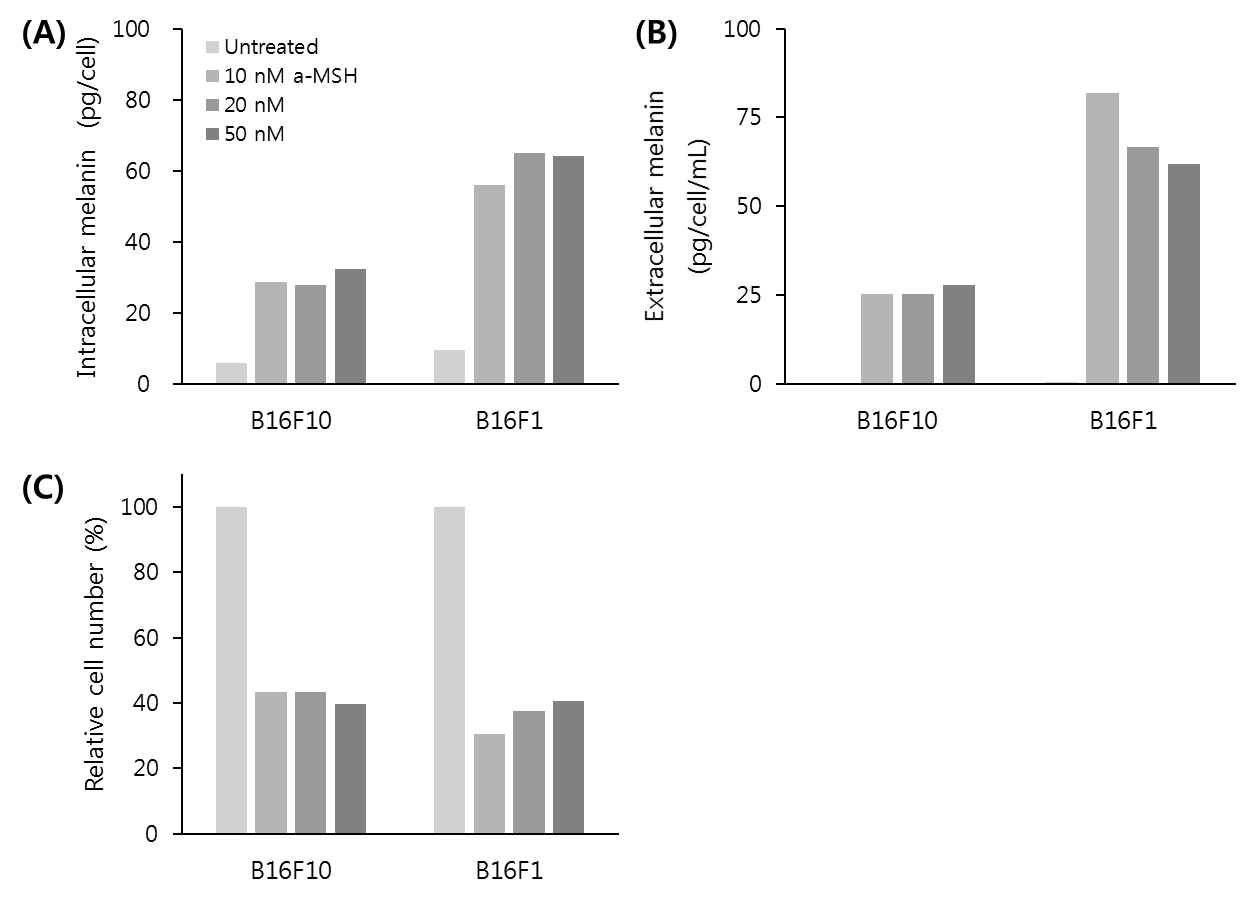


**Figure S1. Effect of α-MSH treatment (0 to 50 nM) on melanin synthesis and proliferation of 2D cultured melanocytes.** Plots for (A) intracellular melanin, (B) extracellular melanin, and (C) relative cell number.


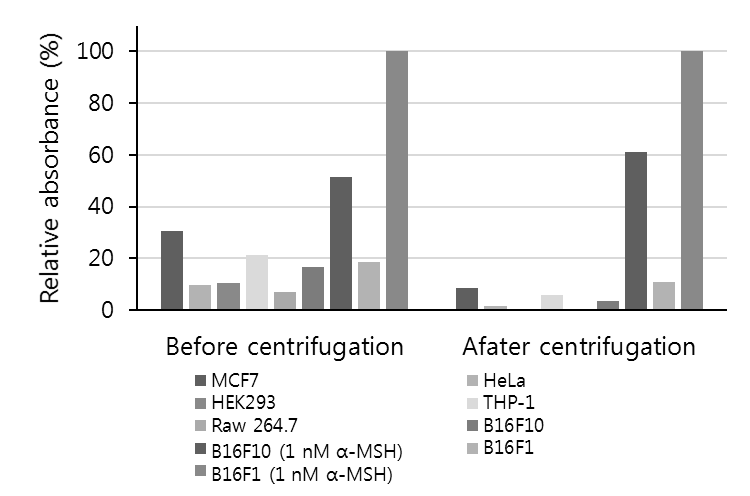


**Figure S2. Relative absorbance of a number of different cell lines and B16 cells.**


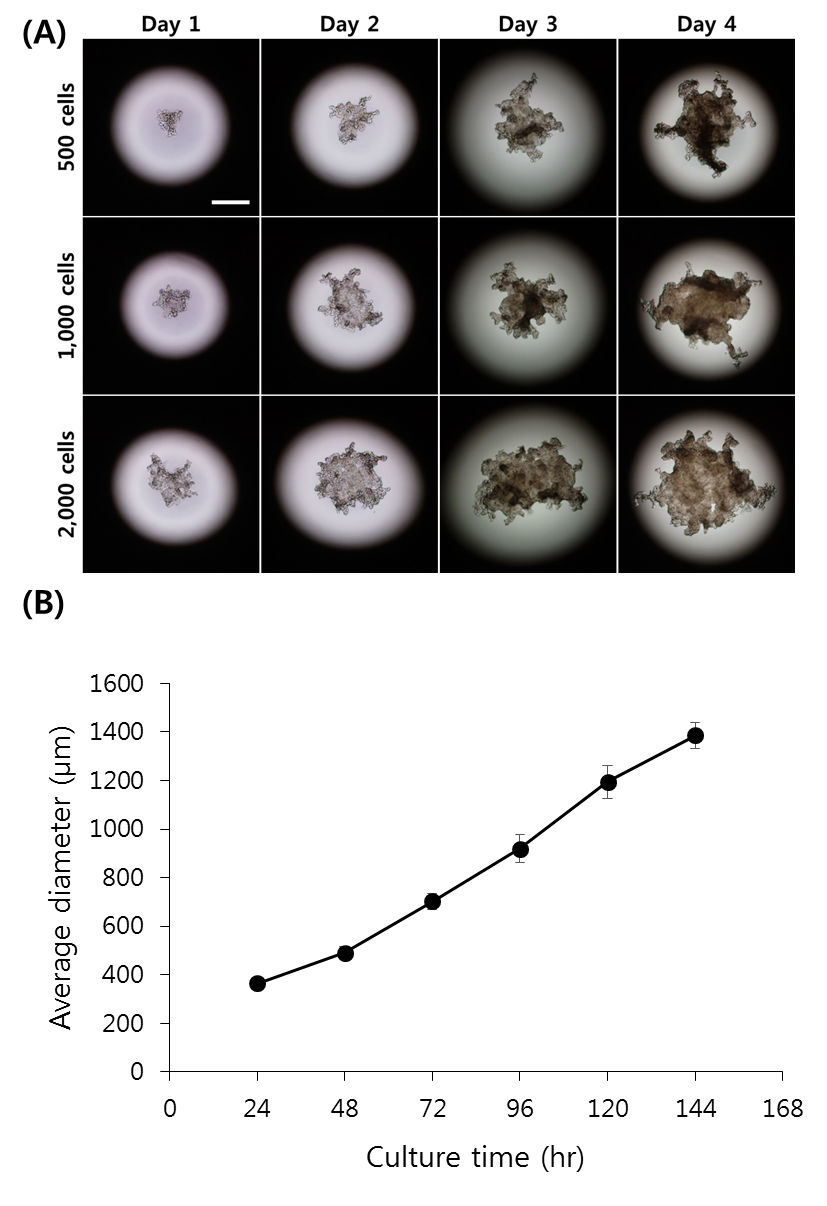


**Figure S3. Growth of B16F10 aggregate in a hanging drop** (A) Daily micrographs of B16F10 aggregates of different starting cell numbers. (B) Growth curve of B16F10 aggregates started with 500 cells (n=4 − 10). Scale bar= 500 µm.


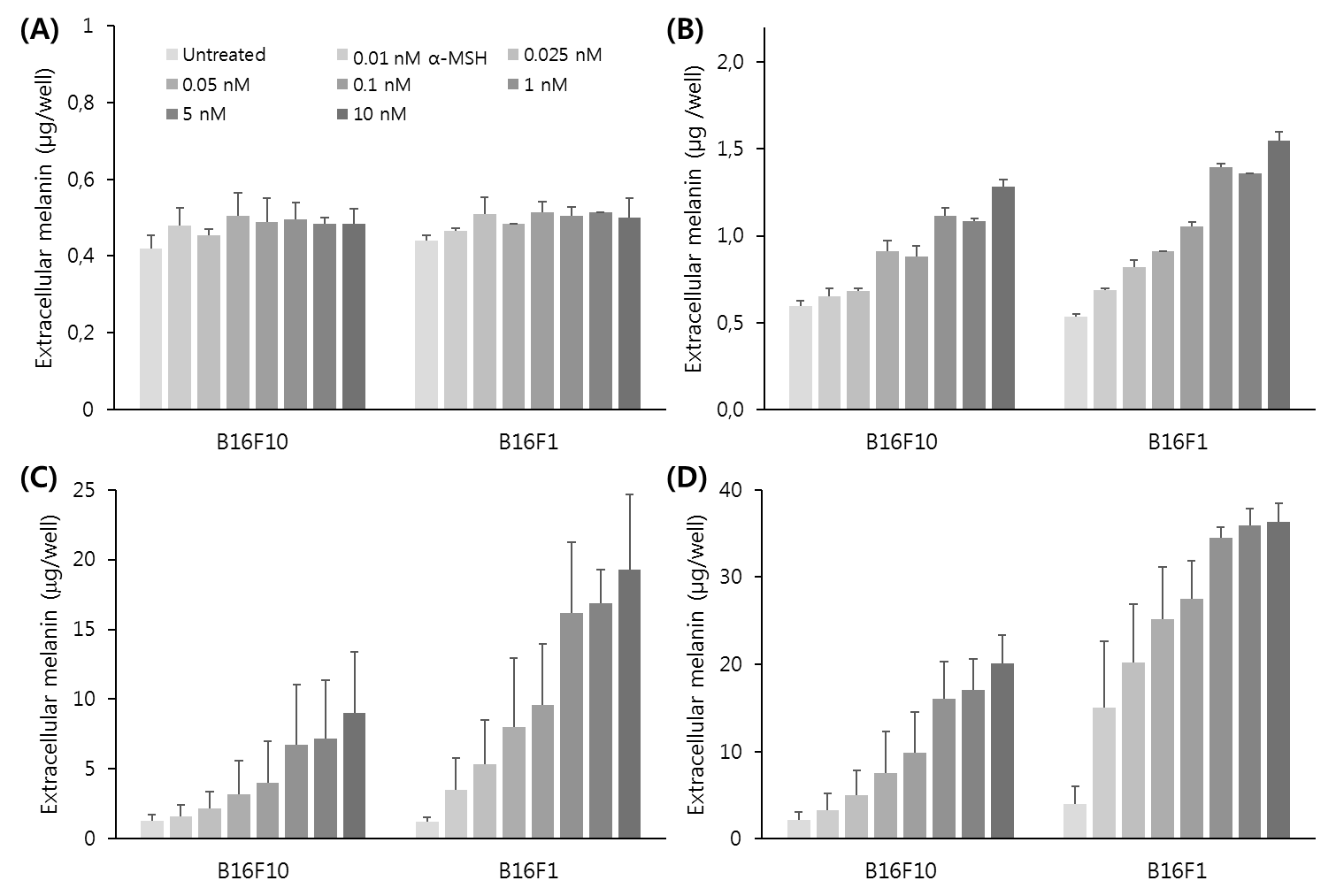


**Figure S4. Effect of α-MSH on the extracellular melanin production of a B16 aggregate.** (A) 24 hours, (B) 48 hours, (C) 72 hours, and (D) 96 hours post transfer.


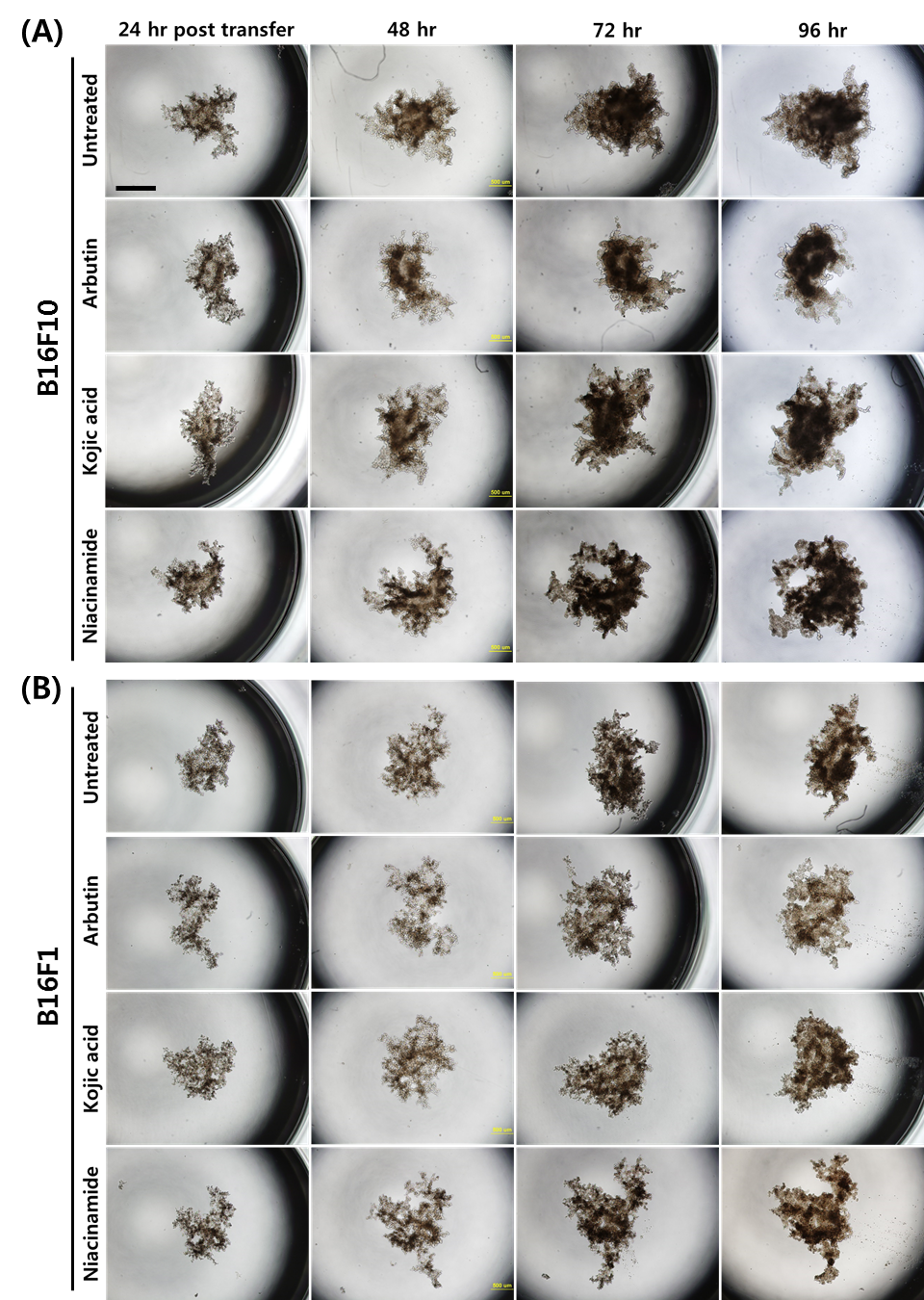


**Figure S5. Time-series micrographs of a melanocyte aggregate treated by depigmenting** **agents** (A) B16F10 and (B) B16F1 cells. Scale bar = 500 µm


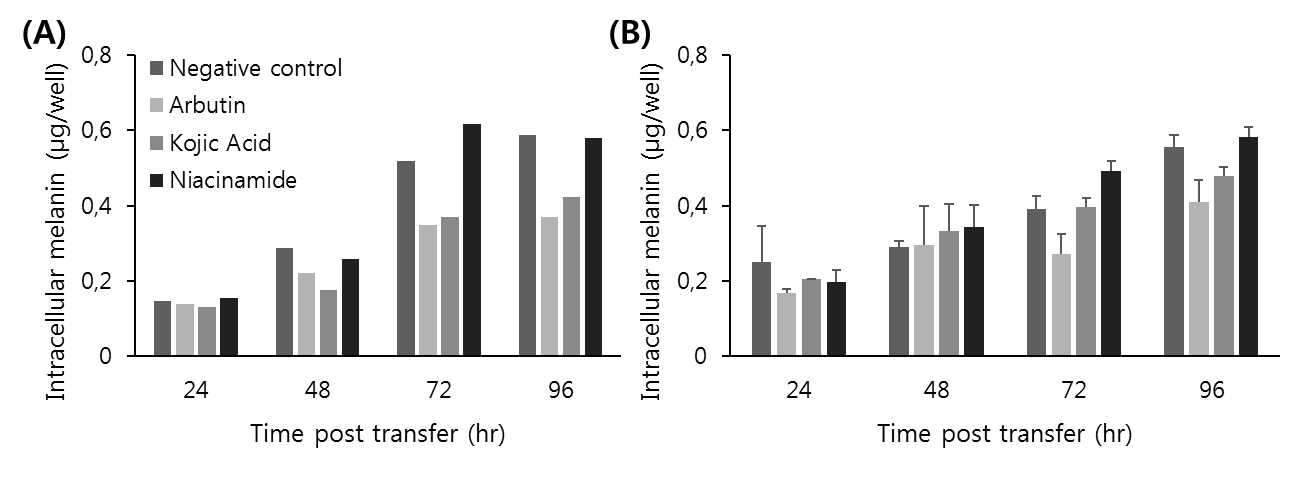


**Figure S6. Depigmenting effects of arbutin, kojic acid, and niacinamide on melanocytes in a 3D culture.** Time-course change intracellular melanin production in (A) B16F10 and (B) B16F1 cells.

**
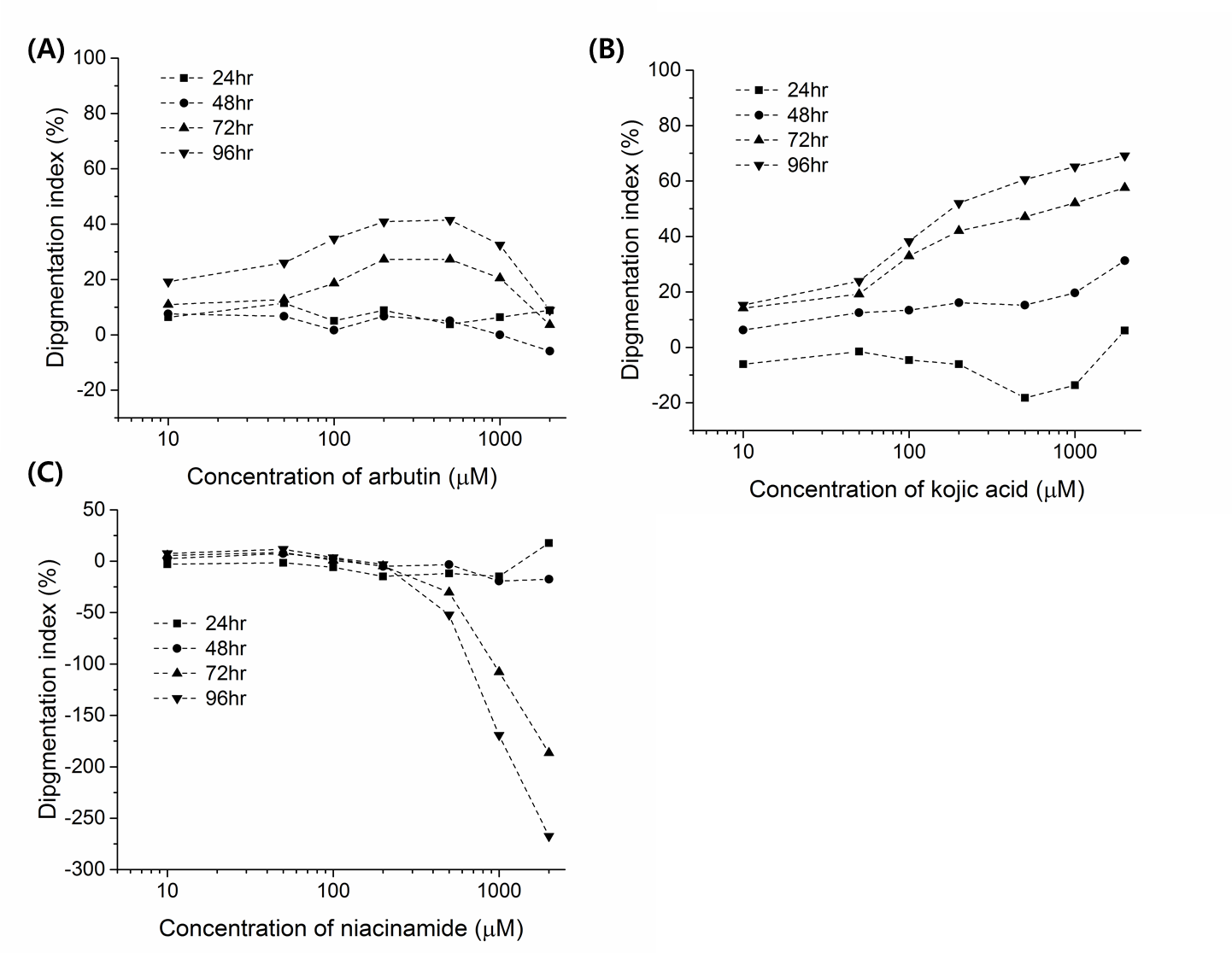
**

**Figure S7. Daily plot of depigmenting index of B16F10 aggregate treated with (A) arbutin, (B) kojic acid, and (C) niacinamide**


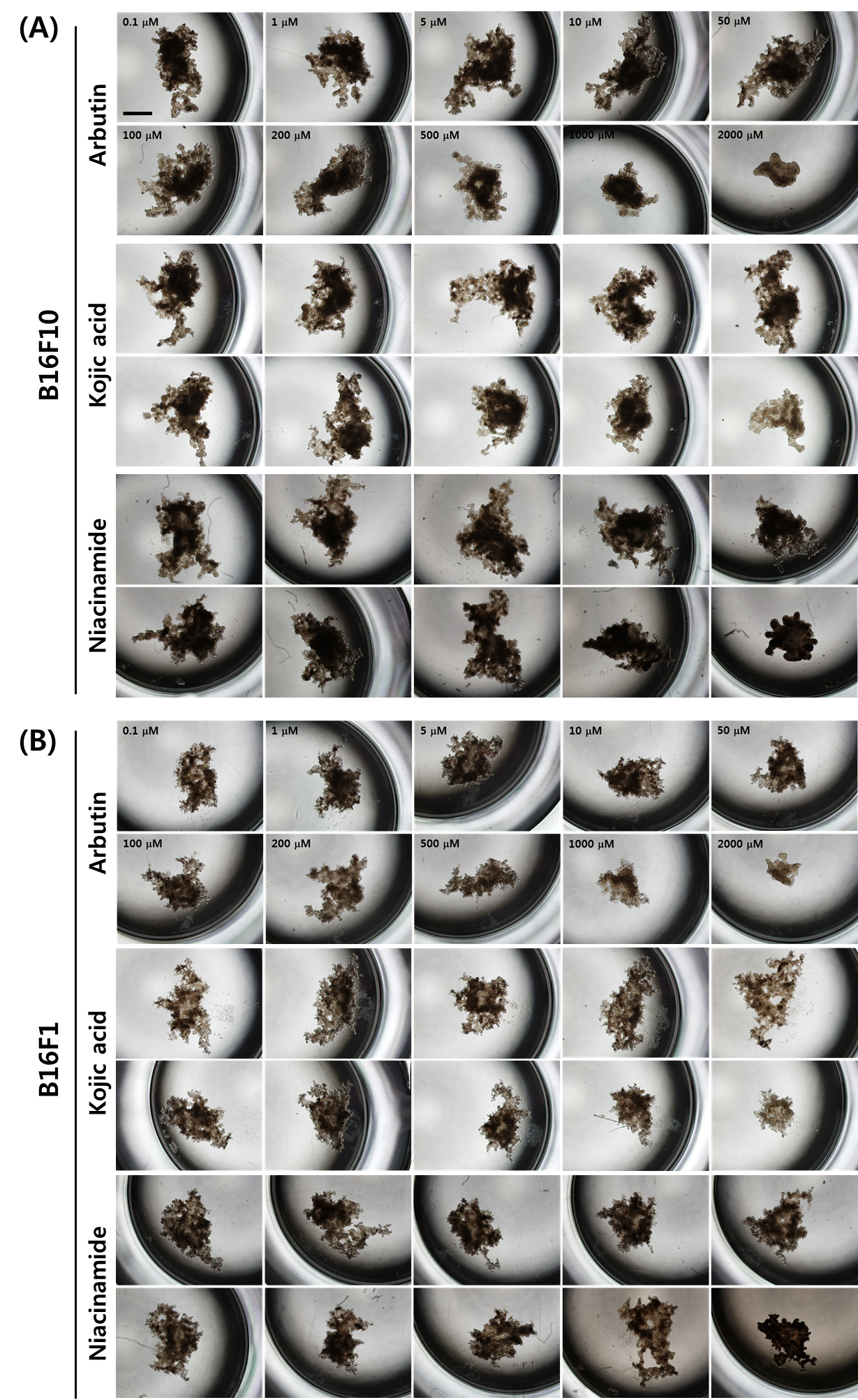


**Figure S8. Micrographs of (A) B16F10 and (B) B16F1 aggregates at 96 hours after the treatment of each depigmenting agent with different concentrations ranging from 0.1 to 2,000 µM.** Scale bar = 500 µm.
